# Ocular Drug Delivery for Glaucoma Management

**DOI:** 10.3390/pharmaceutics4010197

**Published:** 2012-03-08

**Authors:** Nathan Gooch, Sarah A. Molokhia, Russell Condie, Randon Michael Burr, Bonnie Archer, Balamurali K. Ambati, Barbara Wirostko

**Affiliations:** 1 Department of Bioengineering, University of Utah, Salt Lake City, UT, USA; 2 Department of Pharmacy, University of Utah, Salt Lake City, UT, USA; 3 Moran Eye Center, University of Utah, Salt Lake City, UT, USA

**Keywords:** glaucoma, IOP, drug delivery, sustained release, polymer, inserts, punctal plug

## Abstract

Current glaucoma management modalities are hindered by low patient compliance and adherence. This can be due to highly complex treatment strategies or poor patient understanding. Treatments focus on the management or reduction of intraocular pressure. This is most commonly done through the use of daily topical eye drops. Unfortunately, despite effective therapies, glaucoma continues to progress, possibly due to patients not adhering to their treatments. In order to mitigate these patient compliance issues, many sustained release treatments are being researched and are entering the clinic. Conjunctival, subconjunctival, and intravitreal inserts, punctal plugs, and drug depots are currently in clinical development. Each delivery system has hurdles, yet shows promise and could potentially mitigate the current problems associated with poor patient compliance.

## 1. Glaucoma: The Silent Thief of Sight

Glaucoma is a slowly progressive pathology that can result in the loss of peripheral vision, decreased contrast sensitivity, and loss of visual acuity. Due to the asymptomatic nature of the early phases of the disease most patients experience undiagnosed loss of vision until the advanced stages of the disease have occurred. Thus the disease is known as the “silent thief of sight”. This indolent optic neuropathy is characterized structurally by a loss of retinal ganglion cells and optic nerve axons. Glaucoma is the second leading cause of the world’s blindness with nearly 70 million cases worldwide and accounting for 12% of all cases of preventable blindness [1,2,3]. It is estimated that by 2020, close to 4 million Americans will have glaucoma with 50% undiagnosed and approximately 120,000 individuals developing blindness. [4,5].

Glaucoma’s strong correlation with raised intraocular pressure (IOP) has been demonstrated by large prospective randomized trials [6,7,8,9,10,11]. Increased IOP and IOP variability are now recognized as significant risk factors both for the development and the progression of glaucoma, with open-angle glaucoma (OAG) in particular [6,9,10,11]. At present, the majority of OAG treatment modalities focus on the management and reduction of IOP. The standard goal of treatment is to reduce IOP by 20–50% from which damage was sustained. Current pharmacotherapies such as pharmaceutical treatment with eye drops and gels, laser treatment, or incisional surgery achieve a lower IOP by either decreasing aqueous production or improving aqueous outflow [12,13].

In developing countries, where the access to adequate care and therapies is limited, people are going blind from a disease that can be successfully treated. Patients in these countries may not have the ability to get to their clinics routinely for refills and exams [14]. However, even in the US with ready access to medical care and pharmaceuticals, glaucoma continues to progress in many patients [15]. Often poor IOP control is due to poor compliance and adherence to daily topical treatment regimes or inadequate, complex dosing regimens [16]. Despite effective monotherapy agents, data has shown that upwards of 40% of OAG patients require combination therapy for IOP reduction with close to 75% of glaucoma patients requiring adjunctive therapy after five years [7]. The complexity, cost, and administration issues with multiple medications further reduce patient compliance and adherence. Prescribing pharmacy claims data show the vast majority of patients do not take their topical medications or renew their prescriptions, resulting in patients regularly missing doses. Pharmacy records indicate that close to two months can go by between refills even for simple to use once a day prostaglandins analogs (PGAs) [17]. Of patients discontinuing their initially prescribed medication, more than half failed to restart any topical therapy (827/1624 [51%]) in the span of one year [18]. Retrospective population based data suggests a minority of patients consistently adhere to their topical medication [19]. A sustained mode of delivery where the patient’s dependence on daily self instillation is eliminated could dramatically improve these statistics.

Data confirm that many patients are unable to self-administer drops effectively, including the arthritic aging population and uncooperative pediatric glaucoma patients [20]. Patient videos and questionnaires have demonstrated the inability for patients to effectively self dose and administer the drops accurately and as prescribed [21]. Recent data revealed that only 71% of 204 glaucoma patients were able to get a drop into the eye, and only 39% did so without touching the bottle to the surface of the eye [22]. Such studies confirm eye drop wastage, potential contamination of the eye drop bottles, and poor understanding of the situation among participants. 

Side effects of glaucoma medications are also undermining patient compliance [23]. They can range from local minor effects such as redness, dry eye, burning, and foreign body sensation, to more serious systemic effects such as shortness of breath, fatigue, and low blood pressure or heart rate. An alternative delivery mode could substantially improve the local ocular and systemic safety and tolerability profile by decreasing the amount of drug delivered locally thus limiting systemic exposure.

There are currently several novel and innovative sustained release (SR) delivery methods in various stages of development. This paper will review some broad drug delivery platforms, the current landscape for treating glaucoma with these alternative delivery modes, and will discuss what data are needed in development to allow such a novel technology to be a clinically viable marketed product.

## 2. Ocular Drug Delivery

Current innovation in glaucoma treatment is focused on the improvement of drug delivery methods. The aim is to deliver drugs locally in a controlled manner while mitigating the challenge of poor patient adherence, compliance, and persistence. Currently, the patient who is receiving maximal medical therapy may use up to four different classes of topical medications. A medication’s cost, the complexity of a medical regimen, and the side effects of medications are all factors that may contribute to noncompliance. Innovative technologies may address these issues by ultimately leading to better patient outcomes, a better quality of life, and cost savings to society. To develop a viable, reproducible, SR technology one must consider: (1) formulation work; standardizing the release kinetics, and duration of action; (2) clinical study design (determining the timing of replacement or refill, and identifying the acceptable safety risk profile compared to the topical comparator); (3) encouraging physician and patient acceptance of perhaps a more invasive procedure; and (4) navigation of reimbursement issues to establish the rationale of a perhaps more costly product over the generic comparator.

From a technology perspective, the development of IOP lowering SR therapies is one of the fastest growing segments of the glaucoma market. This is due to the abundance of generic IOP lowering agents, first line branded drugs coming off patent, and the need to improve the compliance rates for these conventional therapies [24]. When considering total glaucoma-related pharmaceutical revenues, the market is actually declining due to drugs losing patent protection with subsequent generic competition. Significant revenue opportunities exist for first to market products that are able to show safety and efficacy in lowering IOP with generic SR reformulations. Furthermore, from a clinical and regulatory perspective, these drugs are being repurposed for the same indication (IOP lowering based on local delivery to the target ocular tissue) albeit with novel release delivery profiles and bioequivalency data. Well established safety risk benefit profiles exist for daily topical pharmacotherapies with extensive clinical data thus eliminating much of the concern and evaluation that is associated with a novel chemical entity. First steps in improving adherence and local tolerability could be as simple as changing the formulation thus improving ocular residence time.

When a topical medication is chosen as a first-line therapy for a patient with OAG a stepladder approach is used [25]. Typically monotherapy is attempted before additional agents are added. Medications are selected based on their potential contribution to IOP reduction and the tolerability of their side effects.

There are currently five main classes of topical medications for the treatment of glaucoma. Most pharmacotherapies either decrease aqueous production (beta blockers (BB), alpha agonists (AA), and carbonic anhydrase inhibitors (CAIs)) or improve aqueous outflow (cholinergics, PGAs). There are also combined medications such as BB with AA or CAIs. Topical BB and CAIs are associated with fewer systemic side-effects than their oral forms and are better tolerated by many patients. PGAs have the advantage of effectiveness in lowering IOP with once daily dosing. However, some patients experience an irreversible change in iris color and periorbital dermal darkening with PGAs [26]. Recent research in topical delivery for glaucoma is focused on new drug carriers and formulations that will improve cornea penetration in a SR manner. Many of these approaches include the use of nanospheres, liposomes, and permeability enhancers to work in tandem with the drug formulation.

Conventional eye drops face rapid tear turnover. Only 1–3% of the topical dosage penetrates to target tissues [27]. A wide variety of novel ocular drugs, including nucleic acids such as antisense oligonucleotides and siRNAs, are being investigated in tandem with nanosphere and microsphere ocular drug delivery methods to enhance cellular penetration, protect against degradation, and improve the solubility of normally poorly soluble drugs in order to allow for long-term delivery [28]. Liposomes, are vesicular lipid systems of a diameter ranging between 50 nm and a few micrometers. They provide a convenient way of obtaining slow drug release from a relatively inert depot. Research has shown that drugs within neutrally charged liposomes result in similar IOP reduction and lasted twice as long as the conventional eye drop, suggesting that the liposomes increased the residence time of the drug [29]. This could reduce dosing frequency. Surfactants, bile acids, chelating agents, and preservatives have all been used as permeability enhancers. Cyclodextrins, cylindrical oligonucleotides with a hydrophilic outer surface and a lipophilic inner surface that form complexes with lipophilic drugs, are among the more popular permeability enhancers. They increase chemical stability and bioavailability and decrease local irritation [30].

## 3. Delivery Devices in Preclinical Development

### 3.1. External Pumps

Several innovative technologies are currently in preclinical development. The Replenish, Inc. (Pasadena, CA, U.S.) device consists of a reservoir for IOP lowering medication, a hydrolysis-based pump system and a cannula that delivers the drug into the anterior chamber. The device is designed to be a non-absorbed, semi-permanent, refillable device. It is implanted much like a tube shunt under the conjunctiva. It can be refilled in the office and can be non-invasively tuned to modulate the drug release rate. Clinically, IOP regulating pharmaceuticals have never been administered intracamerally, hence safety studies will need to be conducted prior to clinical use. 

In general, manually and electrically controlled mini drug pumps, like the Replenish device, are designed, fabricated, and tested using principles of microelectromechanical systems (MEMS) engineering [31]. A reservoir can be implanted subconjunctivally and a cannula is then inserted through an incision into either the anterior or posterior segment. Once the drug reservoir is depleted it can be refilled through a check valve (a one way valve), perhaps refilled over months to years. Electrically controlled pumps incorporate implanted batteries into the design. These devices can also drive electrolysis by wireless inductive power transfer. Electrolysis results in the electrochemically induced phase change of water to hydrogen, and oxygen gas generates pressure in the reservoir, forcing the drug through the cannula. Drug delivery is achieved simply by adjusting the applied current. Prototypes of MEMS (*i.e.*, Replenish) with ocular hypotensive agents, 0.5% timolol or 0.004% travoprost, were implanted in two dogs under the temporal conjunctiva with the cannula inserted into the anterior chamber [32]. The reduction of IOP was achieved for 8 hours with no complications observed out to 3 months. Device concerns include the potential of traumatic damage to intraocular structures during implantation, as well as for the risk of endophthalmitis from continued external contact to the anterior chamber from the reservoir.

Replenish, Inc. soon plans to enter trials for FDA approval of a refillable programmable pump that is implanted onto the surface of the globe to deliver IOP lowering agents directly to the trabecular meshwork. The Replenish device is expected to last more than five years before needing replacement [32].

### 3.2. Contact Lenses

Contact lenses are currently in preclinical stages as replaceable drug delivery devices. As a drug delivery vehicle, they are desirable because they are a patient accepted, non invasive, and a relatively safe product. Contact lenses that are commonly used today for vision correction are often composed of poly-2-hydroxyethylmethacrylate (p-HEMA) hydrogels. *In vitro* testing has shown that drug can diffuse from the hydrogel at therapeutic levels for up to 4 days [33]. Furthermore, researchers have shown that p-HEMA can be synthesized in the presence of drug nanoparticles for the purpose of reducing water solubility of drugs and lengthening elution profiles [33]. This is one method of increasing the residence time of drug particles inside the contact lens, thus increasing the duration of continued SR.

In an effort to further increase the duration of therapeutic drug delivery and improve the drug delivery kinetics, other contact lens designs have been attempted such as creating a drug depot in a degradable poly(lactic-co-glycolic) acid (PLGA) and coating the depot in p-HEMA, which is non-degradable [33]. *In vitro* studies of these lenses showed drug release with zero-order kinetics for up to 4 weeks [34,35]. A limitation to the usage of contact lenses as drug delivery devices is that it requires patients to have steady hands and wear the contact lenses at all times possibly limiting the utility of other drugs being administered concurrently and raising safety concerns (e.g., corneal abrasion, neovascularization, and infection).

### 3.3. Sub-conjunctival Injections

Conjunctival or subconjunctival administration of IOP lowering agents with SR for 3–4 months is another attractive alternative to daily eye drops. A time frame of 3–4 months of delivery is consistent with the frequency of routine glaucoma visits. Delivery over this extended time period is theoretically possible considering the volume available in and under the conjunctival space, as well as degradation rates of biocompatible polymers. Injections in this region are minimally invasive and well tolerated by most patients. In spite of the potential advantages of bypassing patient compliance issues and the simplicity of in office administration, to date there are no subconjunctival delivery systems in clinical trials.

The ideal subconjunctival delivery of an IOP lowering drug would allow the drug to maintain suitable stability, be permeable across the sclera, provide SR out to 3–4 months, and minimize systemic and lymphatic absorption. Possibly the most significant challenge is a lack of clinical evidence to date supporting 3–4 months of IOP reduction. Although target *in vitro* SR rates are achievable and continuous IOP reduction has been shown in animal models, it is yet to be seen if this translates to clinical studies [36].

There are currently several technologies in development to achieve targeted release profiles with approved IOP lowering drugs. Timolol maleate has been incorporated into PLGA microparticles with a double water-in-oil emulsion technique [37]. One such formulation of PLGA exhibited SR for over 100 days in vitro with 100% of drug release at this point. Disadvantages to this method are a burst effect of 30% of the total drug after one day and only a 20% loading efficiency. Timolol eye drops have known cardio-pulmonary adverse side effects from excessive systemic absorption and these bursts could potentially lead to systemic adverse events.

Another SR formulation in pre-clinical development is liposomal latanoprost [36]. The formulation, which involves encapsulation of lipophilic latanoprost within a lipid bilayer, was tested *in vivo* in normotensive New Zealand white rabbits. IOP was lowered by 2–3 mmHg when compared to the non-treated rabbits and showed greater IOP reduction than topical latanoprost drops. The IOP reduction from a single injection continued for 50 days at which point another injection was administered and a similar IOP lowering effect was shown out to 80 days. No adverse side effects were recorded even considering that a significant burst effect was suggested, however, the authors did not report any pharmacokinetic data. An advantage of this liposomal formulation is that the excipient benzalkonium (BAK) is not needed considering that the injection is a single use product. BAK is used in many topical multi-dose eye drop formulations as a preservative and has been implicated in ocular surface disease [38]. Products devoid of BAK offer an improved safety profile eliminating this unwanted side effect [39]. A drawback of a liposomal formulation is that the ocular safety and biocompatibility is less established than PLGA and other polymers.

Scleral permeability mechanisms are poorly understood making it difficult to predict a drug’s interaction in the subconjunctival space and subsequent intraocular penetration. Physicochemical properties such as hydrophilicity/lipophilicity, acid/base characteristics, and molecular weight alter a drugs ability to permeate the sclera. Hydrophilic drugs better penetrate the sclera and have more pronounced burst effects where lipophilic drugs generally have smaller burst effects but poor scleral permeability [40,41]. This creates a design paradigm when considering the ideal formulation because both good scleral permeability and a low burst effect are crucial to achieve safety and efficacy clinically. Strategies to minimize burst effects could be through excipient selection and multiple elements of controlled release (*i.e.*, degradation of microspheres and diffusion through a membrane) [42,43,44].

## 4. Delivery Devices in Clinical Development

Several prospective products are already in clinical development attempting to be the first to market as a sustained delivery of a generic IOP lowering agent (Table 1). The precise developmental status of many of these candidates is uncertain as they work through technical and clinical hurdles. Some of these products have encountered challenges with the mode of delivery. These have included such issues as long term device retention, which brings into suspect the ability of the drug to actually get to the ocular tissue continuously. Other challenges such as the insertion process have limited the ability of clinicians to affectively and safely place the device or depot. 

Other opportunities to hasten clinical development incorporate generic drugs with non-proprietary polymers such as PLGA, Polycaprolactone (PCL), and chitosan. These polymers are attractive because their established ocular biocompatibility profiles and well recognized release kinetics can hasten the development path.

**Table 1:** Glaucoma intraocular pressure (IOP) lowering sustained release (SR) platforms that have reached clinical development.

Company	Drug	Delivery Method	Clinical Development	status
pSivida Corp.	Latanoprost	Subconjunctival/Perilimbal	Phase I/II	recruiting
Alcon	Anecortave	Subconjunctival/Subtenons	Phase II/III	discontinued
Allergan	Brimonidine	Intravitreal	Phase II	ongoing
QLT	Latanoprost	Punctal plug	Phase II	completed
Aerie	Latanoprost	Subconjunctival suture fixation	Projected to start Phase I/II in 2012	ongoing

### 4.1. Punctal Plugs

In terms of clinical progress, QLT is the furthest along. Punctal plugs have long been used for the treatment of dry eye syndrome and are a device that could be easily accepted by both patients and physicians. Punctal plugs are tiny, biocompatible devices inserted into the lid puncta to block tear drainage. The use of punctal plugs for the delivery of ophthalmic medications offers a novel approach for chronic treatment of various eye diseases including glaucoma, post operative therapy, and dry eye syndrome. Punctal plugs have several potential advantages over eye drops, including dose reduction, enhanced efficacy, and better patient compliance. Those made from silicone, hydroxyethyl methacrylate, polycaprolactone are intended for 180 day use [45]. While punctal plugs made from animal collagen last for 7–10 days and disintegrate [46]. Recently, punctal plugs made from thermosensitive, hydrophobic acrylic polymer were used to avoid extrusion problems (Cylindrical Smartplug®) and improve retention. This polymer changes from a rigid solid to a soft, cohesive gel when its temperature changes from room temperature to body temperature. Excessive tearing (epiphora) and displacement or loss of plugs are common and can occur for many reasons. Canaliculitis, bacterial build up from punctual occlusion, can also be a concern. Punctal plug drug delivery systems are usually coated with a material that is impermeable to the drug and tear fluid on all sides except the head portion through which the drug is released into the tear film. The release of the drug from a punctal plug is controlled by drug diffusion to the tear fluid. The drug can be in the form of solutions, suspensions, microemulsions, nanoparticles, or liposomes. Some plugs can be soaked in drug solution before insertion; however, these drug-loading approaches, when performed in the outer coat alone, result in limited drug loading. Most punctal plugs have shown near zero-order drug release rates for drug molecules [47,48]. QLT has had some issues with punctal plug retention. Punctal plug-mediated ocular delivery of latanoprost is in Phase II clinical study (QLT, Inc., BC, Canada) with a revised and custom fit plug delivering latanoprost. Recent reports show 60% of subjects at 4 weeks showed an IOP reduction of 5 mmHg or greater with a higher plug retention rate. The IOP lowering efficacy however is still inferior to topically daily administered Xalatan®. 

### 4.2. Conjunctival Inserts

Ophthalmic inserts are sterile preparations, with thin, multilayered, drug-impregnated devices placed into cul-de-sac or conjuctival sac in order to contact the bulbar conjunctiva. Ophthalmic inserts can be divided into three primary categories: soluble, insoluble, and bioerodible. For insoluble inserts, usually the core is a drug reservoir and sandwiched between rate limiting membranes. Ocular inserts allow for controlled sustained release, reduced dosing frequency, and increased contact time with ocular tissue (*i.e.*, better bioavailability). Expulsion and discomfort are the two greatest problems associated with their use.

Ocusert® (ALZA Corporation) is an insoluble insert and was the first ocular sustained release therapy. Ocusert devices are constructed of plastic membranes about one-third the size of a contact lens and inserted into the eye and worn under the upper or lower lid, where they cannot be seen. Ocusert never overcame topical drops as the delivery method of choice because of patient discomfort, the requirement of manual dexterity, patient education requirements regarding device placement and premature device displacement [49].

LACRISERT® (ATON Pharma) is a sterile, translucent, rod-shaped, water soluble, ophthalmic insert made of hydroxypropyl cellulose, for administration into the inferior cul-de-sac of the eye once daily and marketed for dry eye. It reduces the signs and symptoms resulting from moderate to severe dry eye syndromes, such as conjunctival hyperemia, corneal and conjunctival staining. It also has received minimal to moderate market up take. Conceptually, other compounds, such as IOP lowering agents could be formulated in such a device to help address the issues raised in this paper.

### 4.3. Subconjunctival Inserts

Subconjunctival inserts could allow for the use of implants in place of viscoelastic depot delivery injections. The qualities of an ideal subconjunctival injection could be obtained, but with the added risks of performing a minimally invasive surgery as opposed to an in office procedure. pSivida announced a collaboration with Pfizer in mid 2011 to deliver latanoprost via a unique patented drug delivery subconjunctival insert. According to public disclosures they initiated an ongoing Phase I/II study for safety, tolerability and initial efficacy at the University of Kentucky [50]. Aerie Pharmaceuticals has developed a latanoprost ocular insert said to be in preclinical development with plans to be in the clinic in 2012 [51]. An analysis of the company’s publicly available information reveals an ocular insert made by compressing pellets of latanoprost then coating them with a membrane such as ethylene vinyl acetate [52]. This type of insert could provide a solution to the burst effect-hydrophilicity/lipophilicty paradigm with improved ability to control the burst effect through porosity and membrane thickness of hydrophilic drugs that readily permeate the sclera.

### 4.4. Intravitreal Inserts

Topically administered daily ophthalmic brimonidine (Alphagan, Allergan) is indicated for IOP reduction in patients with ocular hypertension and or OAG. Brimonidine works by decreasing the amount of aqueous fluid in the eyes. Generally brimonidine is administered as a topical eye drops 2–3 times per day. Ophthalmic brimonidine has recently been shown to have potential neuroprotective mechanisms in glaucoma patients in addition to IOP lowering. In the Low Tension Glaucoma study, subjects randomized to Alphagan had equal IOP lowering as timolol yet less visual field progression [53]. Neuroprotective treatments are designed to increase cell survival by protecting or enhancing cell injury resistance mechanisms or by helping to inhibit cell death. 

Ozurdex, a degradable dexamethasone intravitreal implant, is used to treat macular edema and noninfectious uveitis. This device is one of two that have been approved by the US Food and Drug Administration (FDA) for intraocular implantation. Ozurdex slowly degrades in the vitreous releasing dexamethasone. Allergan is currently undergoing clinical trials of brimonidine tartrate in the Ozurdex PLGA platform for the treatment of geographic atrophy due to AMD [54]. This device could also translate into glaucoma management as a neuroprotective modality delivering brimondine.

### 4.5. Drug Depots: Anecortave Acetate

Anecortave acetate (AnA), delivered as an anterior subtenons depot for the treatment of elevated IOP (Alcon Laboratories, Inc), showed promise in several initial pilot IOP-lowering studies. This insoluble compound had the benefit of acting as a sustained release suspension when given as a depot for upwards of six weeks. Its angiostatic properties originally were being investigated for wet age-related macular degeneration (AMD), but observations of IOP lowering effect led to glaucoma clinical investigations. Chemical modifications to eliminate the glucocorticoid activity and its mechanism of action were thought to enhance outflow at the trabecular meshwork [55,56]. The hydrophobic molecule resisted diffusion and could therefore create a sustained release depot when injected in the juxtascleral or sub-tenons space.

An uncontrolled prospective case series wherein seven eyes of six subjects were treated with AnA via a juxtascleral delivery method was studied in 2009. Multiple perilimbal injections providing 24 cumulative mg were administered with a 30-gauge needle under topical anesthesia [56]. IOP dropped an average of 9.5 ± 4.5 mmHg within the first week with effective IOP control maintained for a minimum of 3 but up to 19 months. Another study showed that a similar treatment resulted in 34% IOP reduction for one month in 7/8 eyes suffering uncontrolled steroid-induced ocular hypertension (OHT) [56].

Results of Phase II clinical trials were not as encouraging as prospective studies. The safety and efficacy study involved 200 patients who each received one anterior juxtascleral injection. It confirmed sustained IOP lowering at the highest dosage of 60 mg. However the mean reduction was only 3.8 mmHg [57]. In July 2009 Alcon announced that the benefit was insufficient to justify further development [58].

## 5. Conclusions

### 5.1. Market Considerations for the Ideal Delivery Product

Glaucoma is a chronic pathology with few symptoms until late in the disease. Treatment is designed to prevent worsening rather than to improve visual function. Due to the fact that an immediate benefit is not felt by the patient, glaucoma is by nature a disease where compliance is a problem. SR of glaucoma therapies may be an answer to the issues of poor compliance, poor adherence, and even glaucoma progression through better and sustained IOP control. Despite glaucoma medications lowering IOP effectively, they are often unable to flatten the diurnal curve of IOP and if doses are missed due to non compliance longer term IOP control is even worse [59]. With IOP variability and fluctuation both over 24 hours and over the course of weeks to months, glaucoma will most likely progress thus impacting the patient’s visual outcomes. Outcomes data from large multicenter prospective studies support the notion that consistent IOP lowering controlling for variability and fluctuation, may improve visual outcomes [6,60,61]. These SR technologies could show effectiveness in better IOP control both over 24 hours as well as longitudinally thus improving outcomes.

However, efficacy will not be enough to get these products on the market, as there are economic and health care issues that need to be considered. A novel delivery would need to be economically favorable to society and improve upon the standard of care. The U.S. health care system is currently relying on comparative effectiveness data to demonstrate improved quality of life, better outcomes for patients, and more cost effective means of providing care when considering reimbursements [62]. Thus outcomes research and data will need to be gathered during clinical development.

Currently, in the US, eye drops are generally the first choice for treating patients with OAG and generics are reasonably inexpensive. Despite effective therapies, patents progress and it is the cost of potential and future visual impairment resources that has the greatest cost to society. Studies have confirmed that the cost of care for people with glaucoma, over their expected lifetime, is higher than that of people without OAG [63]. Furthermore, with glaucoma progression and more aggressive interventions, the costs increase [64]. Hence therapies that affect outcomes and have the greatest likelihood of slowing progression through better IOP control will have the greatest cost savings to society. 

With the possibility of improved patient outcomes, the argument can then be made that a more expensive drug device would have better long term efficacy, IOP control and less glaucoma progression, thus encouraging payers to reimburse more over the generic standard of care. These results could support a viable reimbursement proposition for a SR commercial product given the significant number of patients with glaucoma on topical IOP lowering therapies that could benefit. The unmet medical need and the potential cost saving to society would cancel out the higher initial upfront cost of the device. 

In summary, the ideal marketed SR drug delivery for glaucoma would have to show comparative efficacy to the topical daily comparator and have an acceptable safety risk profile that would allow it to be accepted by patients and physicians. It would need to demonstrate an advantage over the daily topical generic comparators such as an advantage for long term benefits, *i.e.*, better IOP control, less IOP fluctuation and hence better patient outcomes. It would need to be easy to administer, minimally invasive, and preferably given as an in office procedure every few months. It would have an acceptable safety and efficacy profile and perhaps even decrease the associated local tolerability and systemic safety concerns of the active compound when delivered as a daily topical. 

### 5.2. The Future of Glaucoma Drug Delivery

The incidence of glaucoma is expected to rise dramatically in the next two decades. Thus, there is an urgent need to develop novel ocular delivery systems that meet the poly-pharmacy needs of the population with better patient compliance and better sustained long term IOP control initially. This paper has only focused on treating glaucoma with the current mainstay of therapy; IOP lowering agents. However, it must be recognized that glaucoma is a neurologic condition with retinal ganglion cell loss. Studies have indicated that glaucoma is a complex neurologic disease that affects optic nerves, optic radiations, and the lateral geniculate nucleus as well. Central nervous system (CNS) damage associated with glaucoma has been detected by alterations in optic nerves using magnetic resonance imaging [65]. Since retinal ganglion cell degeneration and vision loss can continue despite IOP normalization some propose that nervous system-based factors can also mediate glaucomatous degeneration. This CNS damage may in itself contribute to the progression of glaucoma raising the opportunity for non IOP focused compounds being delivered in a SR manner and aimed at the neuronal preservation and or protection in glaucoma in the future [66]. 

Regardless of the compound and or mechanism of action, the ideal ocular delivery system is one that achieves and maintains effective drug concentrations at the target site for desired time intervals, minimizes systemic exposure and affords good patient tolerance, acceptance and compliance while maintaining a reasonable quality of life. The challenge as pharmaceutical scientists is to circumvent the protective transport barriers of the eye without inducing undesirable and unattractive side effects and still achieve a safe and effective therapy.

In conclusion, considering what the next steps for glaucoma treatment could be, ophthalmologists, health care providers, and researchers must remember the main therapeutic goal: to prevent or slow vision loss in a patient. Given the recognized high unmet need for improved glaucoma drug delivery, there is much activity, research and excitement in this area. As researchers, we need to look at new delivery technologies in terms of safety and risk, IOP and non IOP efficacy, and development timelines as well as cost. Although various factors and hurdles will continue to play a role in furthering SR development, excitement remains for these novel delivery technologies in the future. 
